# Investigating trajectories linking social cognitive capacity, bias, and social isolation using computational modeling

**DOI:** 10.1093/scan/nsae088

**Published:** 2024-12-19

**Authors:** Szymon Mąka, Marcelina Wiśniewska, Aleksandra Piejka, Marta Chrustowicz, Łukasz Okruszek

**Affiliations:** Social Neuroscience Lab, Institute of Psychology, Polish Academy of Sciences, Warsaw 00-378, Poland; Social Neuroscience Lab, Institute of Psychology, Polish Academy of Sciences, Warsaw 00-378, Poland; Social Neuroscience Lab, Institute of Psychology, Polish Academy of Sciences, Warsaw 00-378, Poland; Social Neuroscience Lab, Institute of Psychology, Polish Academy of Sciences, Warsaw 00-378, Poland; Social Neuroscience Lab, Institute of Psychology, Polish Academy of Sciences, Warsaw 00-378, Poland

**Keywords:** loneliness, social cognition, cognitive bias, drift diffusion modeling, social isolation

## Abstract

Despite theoretical emphasis on loneliness affecting social information processing, empirical studies lack consensus. We previously adopted a clinical science framework to measure the association between social cognitive capacity and bias and both objective and perceived social isolation in nonclinical participants. Our prior study found that while objective social isolation is linked to both social cognitive capacity and social cognitive bias, loneliness is associated only with the latter. This study extended our previous model using a computational approach to capture implicit cognitive processes. We replicated and extended our earlier findings with a new sample of 271 participants, using neuropsychological tasks and a dot-probe paradigm that was analyzed via Drift Diffusion Model. We presented two complementary trajectories of how social cognitive bias may arise: the increased propensity to engage with salient social stimuli or a decreased information processing capacity dependent on the presence or absence of potential social threats. Furthermore, we found evidence that loneliness is associated with the time needed for perceptual processing of stimuli, both directly and indirectly, via social cognitive bias. Taken together, the complex and context-dependent nature of information processing biases observed in the current study suggests that complex and multifaceted interventions should be implemented to counter social information processing biases in lonely individuals.

## Introduction

Loneliness [i.e. perceived social isolation (PSI)] is a subjective state of discrepancy between the quantity or quality of one’s desired and actual social relationships (Perlman and Peplau 1981). Empirical studies have established that loneliness and objective social isolation are distinct psychosocial constructs that exhibit a weak to moderate relationship with each other (Taylor 2020, Okruszek et al. 2021). Some studies indicate that loneliness has the most detrimental effect on mental well-being (Cho et al. 2019, Park et al. 2023), while other researchers emphasize that both phenomena are partially independent risk factors for overall adverse health outcomes (Holt-Lunstad et al. 2015, Ma et al. 2021, Cené et al. 2022, Kelsall-Foreman et al. 2023). Given the fact that loneliness is driven by one’s subjective perception of one’s social relationships, rather than by objective characteristics of one’s social functioning, a lot of attention has been focused on factors that may drive social appraisals in chronically lonely individuals who, according to the currently predominant conceptualization [Evolutionary Theory of Loneliness (ETL); Cacioppo and Cacioppo 2018], may display increased orienting to social cues which may be biased toward social threat hypervigilance. At the same time, empirical support for such mechanisms is rather limited, with studies examining the association between loneliness and social cognitive processes associated with social perception or emotion processing yielding contradictory results (Spithoven et al. 2017). However, as evidenced by previous research in this field, divergent conclusions of the previous studies may be partially accounted for by the methodological factors; for example, the use of *ad hoc* measures with no known psychometric properties and varying conceptualizations of social cognitive processes. Thus, in the largest in sample size up-to-date behavioral study investigating social cognitive mechanisms in loneliness, we have adapted a comprehensive and well-validated battery of neuropsychological tasks (Pinkham et al. 2018) to measure in a psychometrically valid manner the association between social cognitive capacity (SCC) and objective and perceived social isolation in a large cohort of nonclinical participants (Okruszek et al. 2021). This way, we were able to show that while Objective Social Isolation (OSI) is linked both to low-level processing of social cues [as grasped by social perception and emotion recognition Social Cognition Psychometric Evaluation (SCOPE) tasks] and social cognitive bias (SCB), loneliness is associated only with the latter (Okruszek et al. 2021).

While there is general agreement that lonely individuals may show a negative SCB, i.e. systematic tendency to appraise social stimuli in a negative manner, rather than objective reductions or deficits across social cognitive domains, the lack of a clear approach to how to operationalize and measure such a bias may constitute a clear challenge for future studies investigating social information processing in lonely individuals. Importantly, despite the calls to extend the measurement of constructs underlying normal and abnormal behavior by combining multilevel information from genetic, molecular, physiological, behavioral, and self-report data (e.g. RDoC; Morris and Cuthbert 2012), research on SCB rarely goes beyond a single level of explanation to combine multiple units of analysis (Pinkham et al. 2016, Kaurin et al. 2022). Furthermore, the way such a bias is conceptualized is often driven by the field of investigation, e.g. while schizophrenia research focuses on a tendency to interpret ambiguous or neutral social cues as indicative of hostile or aggressive intent from others (Combs et al. 2007, van der Gaag et al. 2013), studies on anxiety disorders tend to investigate perceptual or attentional processes associated with the involuntary tendency toward preferential processing of threatening or negative social stimuli (Cisler and Koster 2010, Pergamin-Hight et al. 2015).

While large-scale inclusion of physiological behavioral markers into loneliness research may not be attainable, one potential way to address the limited reliability of behavioral markers obtained via typical overt measures (i.e. self-report, behavioral accuracies, and reaction times) is to derive implicit behavioral parameters from explicit behavioral data via computational modeling. By formalizing the behavioral outcomes using mathematical models, one may uncover implicit parameters directly linked to specific cognitive systems and avoid caveats associated with analyzing overt outcomes, which may be a juxtaposition of multiple covert factors (Wilson and Collins 2019). One such approach, the Drift Diffusion Model (DDM; Ratcliff and McKoon 2008), has proved to be a particularly promising tool for investigating perceptual and social decision-making processes. The DDM can break down behavioral outcomes from forced-choice action tasks into parameters associated directly with accumulating evidence in favor of one of various options and extraneous sensory or motor processes contributing to an overt behavioral response. This property of the DDM approach has been successfully utilized by Price et al. (2019), who showed that DDM nondecision time has better psychometric properties for studying the impact of social threat on sensory processes in individuals with social anxiety compared to standard behavioral parameters extracted from a dot-probe task. Interestingly, we recently presented preliminary evidence that, compared to nonlonely counterparts, lonely individuals may show a decreased information accumulation rate, as indicated by the DDM drift, rather than an increased susceptibility to the impact of negative social stimuli, as indicated by nondecision time in the dot-probe task (Mąka et al. 2023).

Thus, the aim of the current study is to establish a multilevel model of social information processing in loneliness by replicating our previous findings in a novel cohort of individuals and extending our model by linking overt measures included in it to covert DDM parameters. This way, we can examine whether the previously established link between loneliness and SCB stems from a reduced information processing capacity (Mąka et al. 2023) or an increased susceptibility to the impact of negative social stimuli on socio-perceptual decision-making processes (Price et al. 2019) in lonely individuals.

## Methods

### Participants

Data for the current study were pooled from two projects investigating the neurophysiological underpinnings of loneliness (National Centre of Science, Poland 2018/31/B/HS6/02848 and 2019/35/B/HS6/00517, PI: Ł.O.). Sample 1 consisted of 163 individuals who were recruited to correspond with the distribution of the Revised UCLA Loneliness Scale (UCLA-R) scores in a Polish population. Sample 2 included 108 individuals with UCLA-R scores corresponding to the lowest (Q1) or highest (Q4) scores in a Polish population. In total, 271 right-handed individuals (150 females) aged 18–35 years (M = 24.94, SD = 4.54 y.o.) with no history of substance abuse, cardiovascular or neurological disorders, and, in the case of Sample 2, Magnetic Resonance Imaging contraindications were recruited via social media platforms. Participants were also screened for current depressive episodes as indicated by anhedonia and dysphoria cut-off scores in the Polish version of the revised Center for Epidemiologic Studies Depression Scale (Koziara 2016).

The study procedure was held at the Institute of Psychology PAS in Warsaw. Each participant provided informed written consent to the project-specific procedures, which were the same for each of the projects. The behavioral and self-report procedures described below were approved by the Ethical Committee at the Institute of Psychology, PAS (decisions 21/XI/2019 and 16/VI/2021). A *post-hoc* power analysis, conducted using the “pwr” R package, indicated that a sample size of 271 participants would provide sufficient statistical power (80%) to detect a Pearson correlation coefficient of 0.17.

### Assessment of social cognitive capacity and bias

In alignment with distinctions in clinical neuroscience, we define SCC as the ability to perform information processing functions, typically assessed through performance-based measures related to social perception, emotion recognition, and theory of mind. In contrast, SCB refers to information processing functions that lead to systematically distorted outputs, measured using vignette-based (Ambiguous Intentions Hostility Questionnaire, AIHQ; Combs et al. 2007) and self-assessment questionnaires (Davos Assessment of the Cognitive Biases Scale, DACOBS; van der Gaag et al., 2013) that assess attribution and hostility biases (Roberts and Pinkham 2012). The assessment of SCC in our study was based on tasks recommended by the SCOPE consortium (Pinkham et al. 2018). These tasks, which were either available or previously validated in Polish by our team, have been effectively utilized in studies on social cognitive mechanisms in both clinical (Okruszek et al. 2022) and nonclinical (Okruszek et al. 2021) populations. The battery included four tasks—The Mini Profile of Nonverbal Sensitivity (MiniPONS), the Penn Emotion Recognition Task (PENN ER-40), the Reading the Mind in the Eyes Task (RMET), and the Hinting Task (HT)—covering social perception, emotion processing, and mentalizing processes. Our selection of these social cognition measures was guided not only by their psychometric properties but also by the relative simplicity of adapting these tasks to Polish. Notably, the PENN ER-40 and HT have been highly recommended by Pinkham et al. (2018) for their robust psychometric properties. However, it is important to acknowledge recent critiques of some of these measures. The MiniPONS has faced criticism regarding its psychometric properties, prompting Pinkham and colleagues to recommend caution in its use. Similarly, the psychometric properties of the RMET have been questioned, concerning both its validity and reliability (Higgins et al. 2023). Despite these concerns, in our present sample, all performance-based measures showed correlations with other measures of social cognition, suggesting their continued relevance in capturing various aspects of social cognitive processes. A detailed description of each task may be found in supplementary materials. Descriptive statistics for measures of social capacity and social bias are provided in Table 1. We investigated ceiling effects in SCOPE tasks—they were found only in the case of HTs, with 21 out of 271 (7.7%) participants scoring the maximum possible score on it.

**Table 1.** Summary statistics for measures of SCC and SCB (*N* = 270, one participant was excluded due to an insufficient number of responses in the dot-probe task)

	PENN ER40	PONSS	HINTING	RMET	AIHQ BS	DACOBS42 AB
Mean	82%	47.2	17	26.1	2.7	22.9
SD	8%	4	2.2	3.3	0.6	6.1
Minimum	50%	36	8	14	1	0
Maximum	97%	58	20	34	4	38
Ceiling scores	0	0	21	0	–	–

AIHQ BS, Ambiguous Intentions Hostility Questionnaire Blame Score; DACOBS42 AB, Davos Assessment of Cognitive Biases Scale Attribution Bias subscale; PENN ER-40, Penn Emotion Recognition Task ER-40.

### Assessment of social functioning

In line with our previous research in this area (Okruszek et al. 2021, 2023), the Polish version of the UCLA-R (Kwiatkowska et al. 2017) was used to measure loneliness in participants. The UCLA-R is a 20-item questionnaire with statements about perceived social belonging and isolation. Each item is rated from 1 (Never) to 4 (Often). Higher scores are indicative of more pronounced loneliness. A six-item version of the Lubben Social Network Scale (SNS; Lubben 1988) was used to measure OSI in participants. Two sets of three questions are given to measure the number of relatives and friends, respectively, with whom the participant: (I) is in regular contact, (II) may seek help from, and (iii) may confide in. The main outcome is the sum of the six questions. For the parsimony, the SNS scores have been reversed, so higher scores may reflect a more pronounced OSI. Both of the measures showed a high degree of reliability in the current sample (Cronbach’s α = 0.94 for UCLA-R, α = 0.84 for SNS).

### Dot-probe task

**Figure 1.**
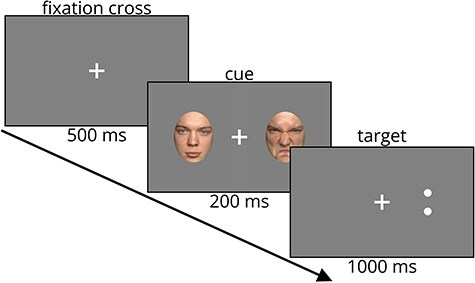
A schema of a trial from the dot-probe task.

Each trial of the task started with the presentation of a white fixation cross for 500 ms, followed by the appearance of two pictures of the same actor placed on either side of the fixation cross (CUE) for 200 ms. After that, a target stimulus (a colon placed either horizontally or vertically, either on the right or on the left side of the fixation cross) was presented for 1000 ms. Participants were instructed to respond by pressing either a right or left arrow key depending on the orientation (vertical or horizontal, respectively) of the colon. The presentation side and orientation of the colon were counterbalanced between trials. The task was presented in two runs of 160 trials each. During the first run, only neutral faces were presented, while in the second run, each trial presented one angry and one neutral face. The face of the same actor was presented twice, once for each block. The facial stimuli consisted of 160 faces of 80 actors, obtained from the FACES database (Ebner et al. 2010) and cropped for the purpose of the current study. The presentation of the neutral/angry stimuli was counterbalanced with regard to the sex of the actors, target positions, and correct response to target. Before starting the main task, participants underwent training, which included 12 trials without cue and 24 full trials. The experimental procedure was programmed using Neurobehavioral Systems Presentation software (version 21.1). The structure of the task is presented in Fig. 1.

### DDM parameters estimation

The preprocessing of behavioral data and estimation of DDM parameters were carried out using R 4.1.1 (R Core Team, 2013). Trials with no behavioral response, responses faster than 200 ms, and reaction times exceeding two standard deviations from the mean for each participant within each condition were excluded from the analysis. One participant was excluded due to an insufficient (<50%) number of responses in the task. The model was set with five free parameters: drift rate (*v*), nondecision time (*t0*), threshold separation (*a*), variability of *t0* (*st0*), and variability in *v* (*sv*). The starting point parameter *z* was set as a threshold divided by two (*a*/2), due to model specification with boundaries representing correct and incorrect responses, respectively. The differential evolution Markov Chain Monte Carlo was used as an estimation procedure based on Hawkins et al. (2017) The convergence of the chains was checked using the Multivariate Potential Scale Factor (MPSF; Brooks and Gelman 1998). The MPSF for all participants was below 1.15, indicating that the chains converged successfully. We calculated *t0* and *v* separately for neutral–neutral (baseline) and neutral–angry conditions. To assess psychometric properties of these measures, an odd–even reliability analysis was conducted using intraclass correlation coefficient—ICC(2,1)—version in Shrout and Fleiss (1979) nomenclature. The code used to estimate DDM parameters may be found at (https://osf.io/7xvfg/). Next, we evaluated whether there was a difference in DDM parameters between dot-probe conditions. A paired samples *t*-test was conducted to compare the scores between the baseline and angry conditions for both nondecision time (*t0*) and drift rate (*v*). For nondecision time (*t0*), there was a significant difference in the scores between the baseline (M = 0.347, SD = 0.039) and angry condition (M = 0.343, SD = 0.039); t(269) = 2.50, *P* = .013. For drift rate (*v*), there was also a significant difference between the baseline (M = 4.098, SD = 0.443) and angry condition (M = 4.003, SD = 0.483); t(269) = 4.80, *P* < .001. Thus, the presence of threatening stimuli decreases the accumulation rate of task-related information, as evidenced by a lower drift rate, while simultaneously reducing reaction time due to nondecisional processes.

### Statistical analysis

In line with the original study (Okruszek et al. 2021), in the first step of the analysis, zero-order correlations were calculated between main social cognition, social functioning, and dot-probe DDM measures. Next, we examined whether the original model incorporating overt measures indicating SCC, SCB, and social isolation was replicated in the sample by combining 271 participants from the current study. For a detailed description of the model, please see Okruszek et al. (2021). Then, we fitted the model by combining 271 participants from the current study with 252 participants from the original (Okruszek et al. 2021) study. This analysis is provided in supplementary materials. Finally, to examine the associations between DDM parameters and overt measures, we examined a new Structural Equation Modeling (SEM) model which includes three types of variable: (i) two outcomes (OSI and PSI), which were entered as two correlated observed variables; (ii) two latent variables corresponding to SCOPE variables [SCC (MiniPONS, PENN ER-40, RMET, Hinting) and SCB (DACOBS42 AB, AIHQ BS)] which were entered as correlated predictors of OSI and PSI; and (iii) two DDM parameters (*t0* and *v*) which, due to their joint modeling, were entered as correlated observed variables and entered as predictors of (i) and (ii) variables. In the SEM approach, exogenous variables—such as drift rate and nondecision time in this context—are typically modeled as correlated by default. This is because, in the absence of predictors, their covariance cannot be explained by other variables in the model. Consequently, their interrelationship remains unexplained. For endogenous variables, while their relationships with exogenous variables are specified, the covariance between these endogenous variables may not be fully accounted for by these relationships alone, suggesting the presence of unexplained covariance due to other potential factors. Since the pair of variables OSI and PSI, as well as SCC and SCB, show stronger correlations with each other compared to their correlations with their respective predictors in the model, we decided to account for their residual covariance by modeling them as correlated within the model.

SEM models were fitted separately for the DDM parameters (*t0* and *v*) extracted from the baseline (Model 1) and neutral–angry (Model 2) trials. We have chosen this approach to account for potential differences in the underlying cognitive processes between the two conditions. By modeling the baseline and neutral–angry trials separately, we aimed to capture condition-specific relationships between the parameters, which may be influenced by the introduction of threatening stimuli. SEM analysis was performed using the Lavaan package (0.6-16), and model fit was assessed using a comparative fit index (CFI > 0.95) and the root mean square error of approximation (RMSEA < 0.06) indices. Statistical inference of model fit was conducted with chi-squared statistics.

## Results

### Correlational analysis

Zero-order correlations from the current sample may be seen in Table 2.

**Table 2.** Zero-order correlations from the current sample.

Variable	Nondecision time baseline	Nondecision time angry	Drift rate baseline	Drift rate angry	AIHQ BS	DACOBS42 AB	PENN ER-40	MiniPONS	RMET	HT	PSI
Nondecision time angry	0.738^***^										
Drift rate baseline	0.213^***^	0.129*									
Drift rate angry	0.195^***^	0.220^***^	0.758^***^								
AIHQ BS	0.077	0.150*	−0.044	−0.013							
DACOBS42 AB	0.003	0.108	−0.145*	−0.030	0.383^***^						
PENN ER-40	0.016	0.039	0.233^***^	0.197^**^	−0.086	−0.148*					
MiniPONS	0.080	0.086	0.162^**^	0.146*	−0.009	−0.212^***^	0.282^***^				
RMET	−0.051	−0.045	0.151*	0.123*	−0.025	−0.084	0.297^***^	0.266^***^			
HT	0.021	0.108	0.085	0.066	−0.013	−0.192^**^	0.161^**^	0.226^***^	0.249^***^		
PSI	−0.122*	−0.063	−0.125*	−0.095	0.367^***^	0.371^***^	−0.138*	−0.194^***^	−0.110	−0.065	
OSI	−0.017	0.057	−0.124*	−0.100	0.198^**^	0.283^***^	−0.161*	−0.238^***^	−0.204^***^	−0.100	0.591^***^

AIHQ BS, Ambiguous Intentions Hostility Questionnaire Blame Score; DACOBS42 AB, Davos Assessment of Cognitive Biases Scale Attribution Bias 42 item subscale; PENN ER-40, Penn Emotion Recognition Task ER-40; PSI, Perceived Social Isolation; OSI, Objective Social Isolation.

* *P* ≤ .05,

** *P* ≤ .01,

*** *P* ≤ .001.

### Model with overt data

In a sample of 270 participants (150 F/120M, 24.9 ± 4.5 y.o.), the original three-factor solution (Okruszek et al. 2021: Lower-Level Social Cue Perception, Higher Level Mentalizing, SCB) was poorly fitted (χ^2^(15) = 47.10, *P* < .001; RMSEA = 0.08; CFI = 0.900). However, the two-factor solution encapsulating all four original SCOPE measures under one latent variable (SCC) provided a good fit to the data (χ^2^(16) =22.51, *P* = .127; RMSEA = 0.03; CFI = 0.98) and was further utilized. The variables included in the model explained over one third of the variance in the PSI (35%) and 22% of the OSI variance.

**Figure 2.**
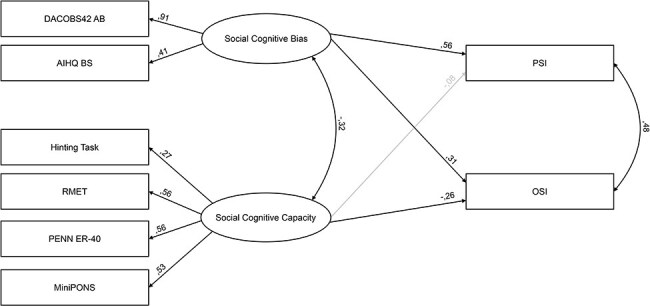
The SEM model with overt data, rectangles depict observable variables, while ellipses symbolize latent factors.

In line with our previous observations, positive correlations were observed between OSI and PSI (r = 0.48 *P* < .001) and negative between SCC and SCB (r = −0.32 *P* < .001). Similarly, in line with our previous report, SCB was linked to both PSI (beta = 0.56, *P* < .001) and OSI (beta = 0.31, *P* < .001), while SCC was a predictor of OSI (beta = −0.26, *P* < .001), but not of PSI (beta = −0.08, *P* = .4). Model is presented on Fig. 2.

### Extended model with DDM parameters

**Figure 3.**
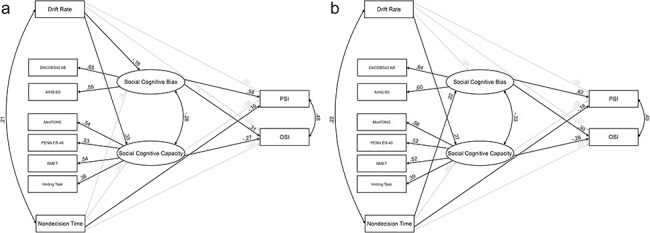
The SEM models of baseline (a; Model 1) and neutral–angry condition (b; Model 2).

Model 1, which is shown in Fig. 3a, had a good fit to the data [χ^2^(24) = 30.35, *P* = .174; RMSEA = 0.031; CFI = 0.982]. Model 2, depicted in Fig. 3b, also exhibited a favorable fit to the data, as evidenced by statistical indices [χ^2^(24) = 32.45, *P* = .116, RMSEA = 0.036, CFI = 0.976]. In each case, a considerable portion of the variability in PSI (Model 1–38%; Model 2–40%) and OSI (22% in both models) was accounted for by the model predictors.

Notably, intercorrelations were found between OSI and PSI (Model 1 r = 0.48, *P* < .001; Model 2 r = 0.49, *P* < .001), *v* and *t0* (Model 1 r = 0.21, *P* = .001; Model 2 r = 0.22, *P* < .001), and SCC and SCB (Model 1 r = −0.28, *P* = .017; Model 2 r = −0.33, *P* = .006).

DDM parameters were significantly linked to overt social cognitive outcomes: in both models *v* was found to be linked to SCC (Model 1 beta = 0.33, *P* < .001; Model 2 beta = 0.27, *P* = .002). Furthermore, *v* in the baseline trials (Model 1) was also weakly associated with SCB (beta = −0.18, *P* = .032). In neutral–angry trials (Model 2), *t0* was found to predict SCB (beta = 0.22, *P* = .008).

In line with our analysis of overt data from the pooled set of 523 participants, SCB emerged as a robust predictor of both PSI (Model 1: beta = 0.58, Model 2: beta = 0.62, *P* < .001) and OSI (Model 1: beta = 0.31, *P* = .001; Model 2: beta = 0.30, *P* = .002), while SCC was negatively associated only with OSI (Model 1: beta = −0.27, *P* = .006; Model 2 beta = −0.26, *P* = .007).

No direct association between *v* and outcome variables was observed. At the same time, *t0* showed a weak negative association with PSI (Model 1: beta = −0.16, *P* = .007; Model 2: beta = −0.18, *P* = .005).

Given the associations between DDM parameters and SCC/SCB, we also investigated indirect effects and found that *v* is indirectly negatively linked to OSI through SCC (Model 1 beta = −0.09, *P* = .016; Model 2 beta = −0.07, *P* = .029), and, in the case of Model 2, *t0* is indirectly positively linked to OSI through SCC (beta = 0.70, *P* = .038). Finally, a positive relationship between *t0* and PSI was found through SCB for negative-angry trials (beta = 0.14, *P* = .014), thus implying the presence of a suppressing effect in Model 2.

## Discussion

The purpose of the current study was to extend the model of trajectories linking social cognitive mechanisms with social isolation initially presented by Okruszek et al. (2021). We used computational modeling to analyze a well-established social information processing task. This allowed us to introduce parameters that signify implicit processes associated with social information processing.

In the first step of the analysis, we corroborated our initial findings by showing that loneliness is linked to SCB, but not to SCC, both in the novel replication sample of 271 nonclinical individuals and in the pooled sample of 523 individuals. Given the clear two-factor structure of social cognitive measures observed in the current data, the findings provide robust evidence that while objective social isolation is linked to objective SCC, no such link can be found for subjective feelings of loneliness. At the same time, SCB, as measured by specific tendencies to appraise others’ actions and intentions in a self-threatening manner, may be linked to both objective social isolation and subjective perception of one’s relationships as lacking.

Secondly, we investigated the trajectories linking overt social cognitive outcomes with implicit processes indicated by DDM parameters. In line with previous findings highlighting the role of the DDM drift rate (*v*) as a reliable marker of perceptual learning processes (Liu and Watanabe 2012), working memory and reasoning (Schmiedek et al. 2007), and cognitive control (Spangler et al. 2022), we found a positive association between drift rate (*v*) and SCC in participants. A more complex trajectory was, however, observed for the association between DDM parameters and SCB: in line with previous reports suggesting that nondecision time (*t0*) may be linked to bias measures in clinical anxiety (Price et al. 2019), we found a positive relationship between nondecision time (*t0*) in threat-related trials and SCB in participants. However, when no threat was present (neutral–neutral block of trials), participants’ SCB was predicted by their information processing capacity, as indicated by drift rate (*v)*, not by nondecision processes, including attentional engagement with social stimuli, as indicated by nondecision time (*t0*).

By its very definition, cognitive bias may be defined as a “systematic error in judgment and decision-making (…) which can be due to cognitive limitations, motivational factors, and/or adaptations to natural environments” (Mata 2012, p. 531). Thus, we hypothesize that, while in the presence of threat-related stimuli, high levels of SCB may reflect the tendency to be more captured by salient stimuli; under no-threat circumstances it may simply reflect participants’ tendency to use simplified heuristics in social situations due to their reduced social information processing capacity.

Finally, by introducing DDM parameters into the model, we were able to link objective and perceived social isolation with latent cognitive processes signified by such parameters. First, we found an indirect relationship linking information processing capacity, as indicated by drift rate (*v*), with objective social isolation via SCC, which may indicate a bilateral association between information capacity and actual opportunities for social interaction. Furthermore, a two-fold relationship between loneliness and DDM parameters was found. First, loneliness was negatively linked to nondecision time (*t0*), which suggests that participants with higher levels of chronic loneliness may exhibit facilitated processing of social stimuli independently of its salience. However, in the absence of a nonsocial control task, it cannot be concluded whether this effect represents an increased orienting specifically toward social stimuli, which could be congruent with evolutionary accounts of loneliness (Cacioppo and Cacioppo 2018) or generalized alternations of perceptual decision-making mechanisms in lonely individuals. Secondly, the opposite indirect effect, with PSI being positively linked to nondecision time (*t0*) via SCB, was also found in the presence of threat-related stimuli. This finding suggests that two opposite-direction effects may link PSI with nondecision time (*t0*) in the presence of negative social stimuli, which may account for previous contradictory findings regarding the association between loneliness and attentional bias to threats (Spithoven et al. 2017).

Taken together, the current findings provide a robust and replicable model linking social isolation variables with social cognitive mechanisms in nonclinical participants. Using a computational modeling approach in loneliness research, we were able to differentiate between implicit processes associated with information processing efficiency and nondecision processes associated with vigilance toward salient stimuli. This way, we were able to provide two complementary accounts of how SCB may arise either due to the increased propensity to engage with salient social stimuli or to decreased information processing capacity dependent on the presence or absence of potential social threats. Finally, we provided evidence that loneliness is associated with nondecision time, both directly and indirectly, via SCB. Importantly, we demonstrated that in the presence of social threats, these two associations have opposite effects, resulting in suppression. Explicit behavioral or self-report measures can be thus insufficient to fully grasp the cognitive mechanisms of loneliness.

Still, several limitations of the current study should be noted: first, due to the construction of our sample and inclusion of a subgroup of participants with either very low or very high loneliness scores, the distribution of the loneliness may not be fully representative of the general sample. Secondly, methodological concerns have been raised with regard to the use of the dot-probe paradigm to study attentional processes (Kappenman et al. 2014); thus, the current results should be replicated across different paradigms. Finally, given the wealth of literature on neural bases of attentional bias, the current investigation could be extended to examine the extent to which DDM parameters are linked with actual neural processes underlying cognitive computations (Price et al. 2019, Mąka et al. 2023).

## Supplementary Material

nsae088_Supp

## Data Availability

Data and code used in this study are available on the OSF repository at https://osf.io/7xvfg/.
