# Supplementary material for: Investigating trajectories linking social cognitive capacity, bias, and social isolation using computational modeling
Source: Soc Cogn Affect Neurosci. 2024 Dec 19;20(1):nsae088. doi: 10.1093/scan/nsae088 (PMC11756555; doi:10.1093/scan/nsae088)
Supplement: nsae088_Supp [file nsae088_supp.zip › New folder/scan-24-139-File007.docx]

SUPPLEMENTARY MATERIALS:

**Supplementary methods:**

1) Social perception was measured with The Mini Profile of Nonverbal Sensitivity (MiniPONS; Ambady & Gray, 2002) - stimuli consisted of 64 two-second vignettes presenting expressions communicated through facial expressions, vocal intonations or gestures of a single female actor; participant was asked to choose a more accurate description of the situation out of two displayed options.

2) Emotion recognition was measured with a) Penn Emotion Recognition Task ER-40 (PENN ER-40; Gur et al., 2002) – the participant had to match each of the 40 presented facial stimuli with the label of emotion expressed in the photo by choosing one out of five options (fear, anger, happiness, sadness or a neutral expression); b) Reading Mind in Eyes Task (RMET; Baron-Cohen, Wheelwright, Hill, Raste, & Plumb, 2001) – based on 36  black-and-white cropped photos of the eye region, the participant had to choose which one out of four proposed adjectives best describes the current mental state of the presented person.

3) Theory of mind was assessed with the use of the Hinting Task (Krawczyk et al. 2020) – for each of ten read-aloud short two-character stories, the participant was asked to infer the implied intentions of one of the characters; in case of an incorrect answer, the participant was presented with an additional hint.

In the Ambiguous Intentions Hostility Questionnaire (AIHQ; Combs, Penn, Wicher, & Waldheter, 2007) the participant read five short stories describing ambiguous social situations and was asked to assess the (i) intentionality, (ii) hostility and (iii) blame of the character's actions towards them, the Blame Score was obtained by averaging the subscale scores.

The Attribution Bias subscale from Davos Assessment of Cognitive Biases Scale (DACOBS AB; van der Gaag et al., 2013) and entails four items which refer to the generalized perception of hostility. While in the original study (Okruszek et. al, 2022) we used an 18-item short version of DACOBS AB subscale, the current study extended this part of the assessment by using the AB subscale from the 42-item full version of the DACOBS questionnaire.

**DDM estimation assumptions**:

The parameters’ prior distributions were established as: a truncated normal distribution with mean 0 and standard deviation 2 (with -5 and 5 as lower and upper limits) for v, a beta distribution with shape parameters 1 and 1 for t0 and st0, and a truncated normal distribution with mean 1 and standard deviation 1 (with 0 and 5 as lower and upper limits) for a and sv.

Sixteen chains for each participant and each condition were run, with a differential evolution probability of 0.05 for a burn-in interval of 20,000 iterations, after which the probability of migration was reduced to zero and the chains were run for a further 20,000 iterations to obtain the posterior sample.

**Supplementary results:**

Reliability analysis: Intraclass correlation coefficients were computed to assess the reliability of Drift Diffusion Model parameters and were found to be within the range of acceptable (Cicchetti, 1994). Specifically, for non-decision time, the intraclass correlation coefficients were ICC(2,1) = 0.836 for the baseline condition and ICC(2,1) = 0.858 for the angry condition. For drift rate, the corresponding coefficients were ICC(2,1) = 0.693 for the baseline condition and ICC(2,1) = 0.719 for the angry condition.

We recreated our analysis of overt model (without DDM parameters) by combining a sample of 271 participants from the current study and 252 participants from the original (Okruszek et al., 2021). We examined differences between measures in the current and previous (Okruszek et al., 2021) samples with descriptive statistics (Table 2, SM) and t-tests (Table 3, SM). All comparisons, except for UCLA-R and SNS, were non-significant. Regarding social functioning measures, UCLA-R showed higher scores and SNS lower scores in the current sample. This difference is likely due to the oversampling of lonely participants, due to the inclusion criteria for the studies which were included in the current study. Zero order correlations from joined data from the current and previous samples may be found in the Table 2 (SM).

| Variable | Current sample (n=271) | Original sample (n=252) | Test statistic | p - value |
| --- | --- | --- | --- | --- |
| Age | 24.9 +/- 4.5 | 25.8 +/- 6.2 | 1.7 | 0.15 |
| Sex | 0.45 +/- 0.5 | 0.47 +/- 0.5 | 0.25 | 0.62 |
| Lubben Social Network Score | 14.9 +/- 5.6 | 16.3 +/- 5.5 | 2.8 | <.01** |
| UCLA Loneliness Scale Revised | 42 +/- 12.6 | 38.5 +/- 10 | -3.5 | < .001*** |
| PENN ER-40 | 83% +/- 8% | 83% +/- 7% | 1.4 | 0.15 |
| PONSS | 47.2 +/-  4 | 47 +/- 4.8 | -0.5 | 0.58 |
| Hinting Task | 17 +/- 2.2 | 17.2 +/- 1.8 | 1 | 0.3 |
| Reading Mind in the Eyes | 26.1 +/- 3.5 | 26.7 +/-  3.5 | 1.8 | 0.07 |
| AIHQ (BS) | 2.7 +/- 0.6 | 2.8 +/-  0.7 | 1.2 | 0.2 |
| DACOBS (AB18) | 11.5 +/- 5.2 | 11 +/- 4.2 | -1.4 | 0.17 |

Table 1, SM. Descriptive statistics for the previous and current samples. Test statistics for comparisons are t-tests for all variables except Age, where a chi-square test was used.

| Variable | AIHQ BS | DACOBS18 AB | PENN ER-40 | MiniPONS | RMET | Hinting Task | PSI |
| --- | --- | --- | --- | --- | --- | --- | --- |
| DACOBS18 AB | 0.375^***^ | - |  |  |  |  |  |
| PENN ER-40 | 0.007 | -0.112^*^ | - |  |  |  |  |
| MiniPONS | -0.018 | -0.248^***^ | 0.300^***^ | - |  |  |  |
| RMET | -0.035 | -0.130^**^ | 0.330^***^ | 0.274^***^ | - |  |  |
| Hinting Task | -0.009 | -0.074 | 0.157^****^ | 0.129^**^ | 0.176^***^ | - |  |
| PSI | -0.271^***^ | 0.537^***^ | -0.096^*^ | -0.145^***^ | -0.103^*^ | -0.049 | - |
| OSI | 0.171^***^ | 0.435^***^ | -0.180^***^ | -0.203^***^ | -0.201^***^ | -0.074 | 0.576^***^ |

* p < .05, ** p < .01, *** p < .001

Table 2, SM. Zero order correlations from joined data from current and previous samples. AIHQ BS - Ambiguous Intentions Hostility Questionnaire Blame Score. DACOBS18 AB - Davos Assessment of Cognitive Biases Scale Attribution Bias 18 item subscale. PENN ER-40 - Penn Emotion Recognition Task ER-40. MiniPONS - The Mini Profile of Nonverbal Sensitivity. RMET - Reading Mind in Eyes Task. PSI - Perceived Social Isolation. OSI - Objective Social Isolation.

In a full sample of 523 participants (284F/239M, 25.3+/-5.4 y.o.), the original three factor solution (Okruszek et al., 2021: Lower-Level Social Cue Perception, Higher Level Mentalizing, Social Cognitive Bias) was poorly fitted (χ²(15) = 43.30, p < .001; RMSEA = 0.060; CFI = 0.959). However, the two-factor solution encapsulating all four original SCOPE measures under one latent variable (Social Cognitive Capacity) provided a good fit to the data (χ²(16) =19.669, p = .235; RMSEA = 0.021; CFI = 0.995) and was further utilized. The variables included in the model explained over one third of the variance of the PSI (35%) and 27% of the OSI variance. In line with our previous observations, positive correlations were observed between OSI and PSI (r = .42 p < .001) and negative between Social Cognitive Capacity (SCC) and Social Cognitive Bias (SCB) (r = -.31 p < .001). Similarly, in line with our previous report, in the pooled sample of 523 participants, SCB was linked to both PSI (beta = .58, p < .001) and OSI (beta = .41, p < .001), while SCC was a predictor of OSI (beta = -.22, p < .001), but not of PSI (beta = -.02, p = .68).


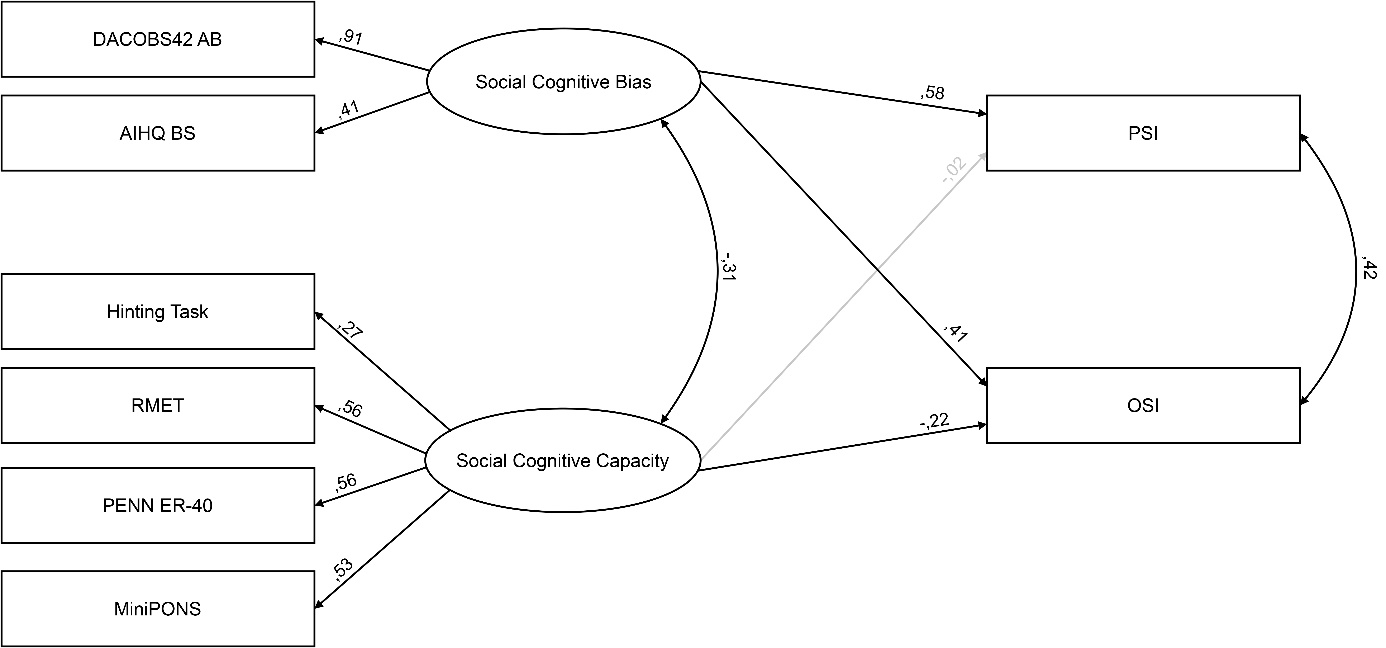
Figure 1 SM. The SEM model fitted on previous and current samples. Rectangles depict observable variables, while ellipses symbolize latent factors. Single-sided arrows denote either factor loadings or regression, as detailed in the text. Double-sided arrows represent correlation.
