# Supplementary material for: Investigating trajectories linking social cognitive capacity, bias, and social isolation using computational modeling
Source: Soc Cogn Affect Neurosci. 2024 Dec 19;20(1):nsae088. doi: 10.1093/scan/nsae088 (PMC11756555; doi:10.1093/scan/nsae088)
Supplement: nsae088_Supp [file nsae088_supp.zip › New folder/scan-24-139-File008.docx]

| Variable | Current sample (n=271) | Original sample (n=252) | Test statistic | p - value |
| --- | --- | --- | --- | --- |
| Age | 24.9 +/- 4.5 | 25.8 +/- 6.2 | 1.7 | 0.15 |
| Sex | 0.45 +/- 0.5 | 0.47 +/- 0.5 | 0.25 | 0.62 |
| Lubben Social Network Score | 14.9 +/- 5.6 | 16.3 +/- 5.5 | 2.8 | <.01** |
| UCLA Loneliness Scale Revised | 42 +/- 12.6 | 38.5 +/- 10 | -3.5 | < .001*** |
| PENN ER-40 | 83% +/- 8% | 83% +/- 7% | 1.4 | 0.15 |
| PONSS | 47.2 +/-  4 | 47 +/- 4.8 | -0.5 | 0.58 |
| Hinting Task | 17 +/- 2.2 | 17.2 +/- 1.8 | 1 | 0.3 |
| Reading Mind in the Eyes | 26.1 +/- 3.5 | 26.7 +/-  3.5 | 1.8 | 0.07 |
| AIHQ (BS) | 2.7 +/- 0.6 | 2.8 +/-  0.7 | 1.2 | 0.2 |
| DACOBS (AB18) | 11.5 +/- 5.2 | 11 +/- 4.2 | -1.4 | 0.17 |

Table 1, SM. Descriptive statistics for the previous and current samples. Test statistics for comparisons are t-tests for all variables except Age, where a chi-square test was used.
