# Supplementary material for: Investigating trajectories linking social cognitive capacity, bias, and social isolation using computational modeling
Source: Soc Cogn Affect Neurosci. 2024 Dec 19;20(1):nsae088. doi: 10.1093/scan/nsae088 (PMC11756555; doi:10.1093/scan/nsae088)
Supplement: nsae088_Supp [file nsae088_supp.zip › New folder/scan-24-139-File009.docx]

| Variable | AIHQ BS | DACOBS18 AB | PENN ER-40 | MiniPONS | RMET | Hinting Task | PSI |
| --- | --- | --- | --- | --- | --- | --- | --- |
| DACOBS18 AB | 0.375^***^ | - |  |  |  |  |  |
| PENN ER-40 | 0.007 | -0.112^*^ | - |  |  |  |  |
| MiniPONS | -0.018 | -0.248^***^ | 0.300^***^ | - |  |  |  |
| RMET | -0.035 | -0.130^**^ | 0.330^***^ | 0.274^***^ | - |  |  |
| Hinting Task | -0.009 | -0.074 | 0.157^****^ | 0.129^**^ | 0.176^***^ | - |  |
| PSI | -0.271^***^ | 0.537^***^ | -0.096^*^ | -0.145^***^ | -0.103^*^ | -0.049 | - |
| OSI | 0.171^***^ | 0.435^***^ | -0.180^***^ | -0.203^***^ | -0.201^***^ | -0.074 | 0.576^***^ |

* p < .05, ** p < .01, *** p < .001

Table 2, SM. Zero order correlations from joined data from current and previous samples. AIHQ BS - Ambiguous Intentions Hostility Questionnaire Blame Score. DACOBS18 AB - Davos Assessment of Cognitive Biases Scale Attribution Bias 18 item subscale. PENN ER-40 - Penn Emotion Recognition Task ER-40. MiniPONS - The Mini Profile of Nonverbal Sensitivity. RMET - Reading Mind in Eyes Task. PSI - Perceived Social Isolation. OSI - Objective Social Isolation.
