# Supplementary figures and images for: Investigating trajectories linking social cognitive capacity, bias, and social isolation using computational modeling
Source: Soc Cogn Affect Neurosci. 2024 Dec 19;20(1):nsae088. doi: 10.1093/scan/nsae088 (PMC11756555; doi:10.1093/scan/nsae088)

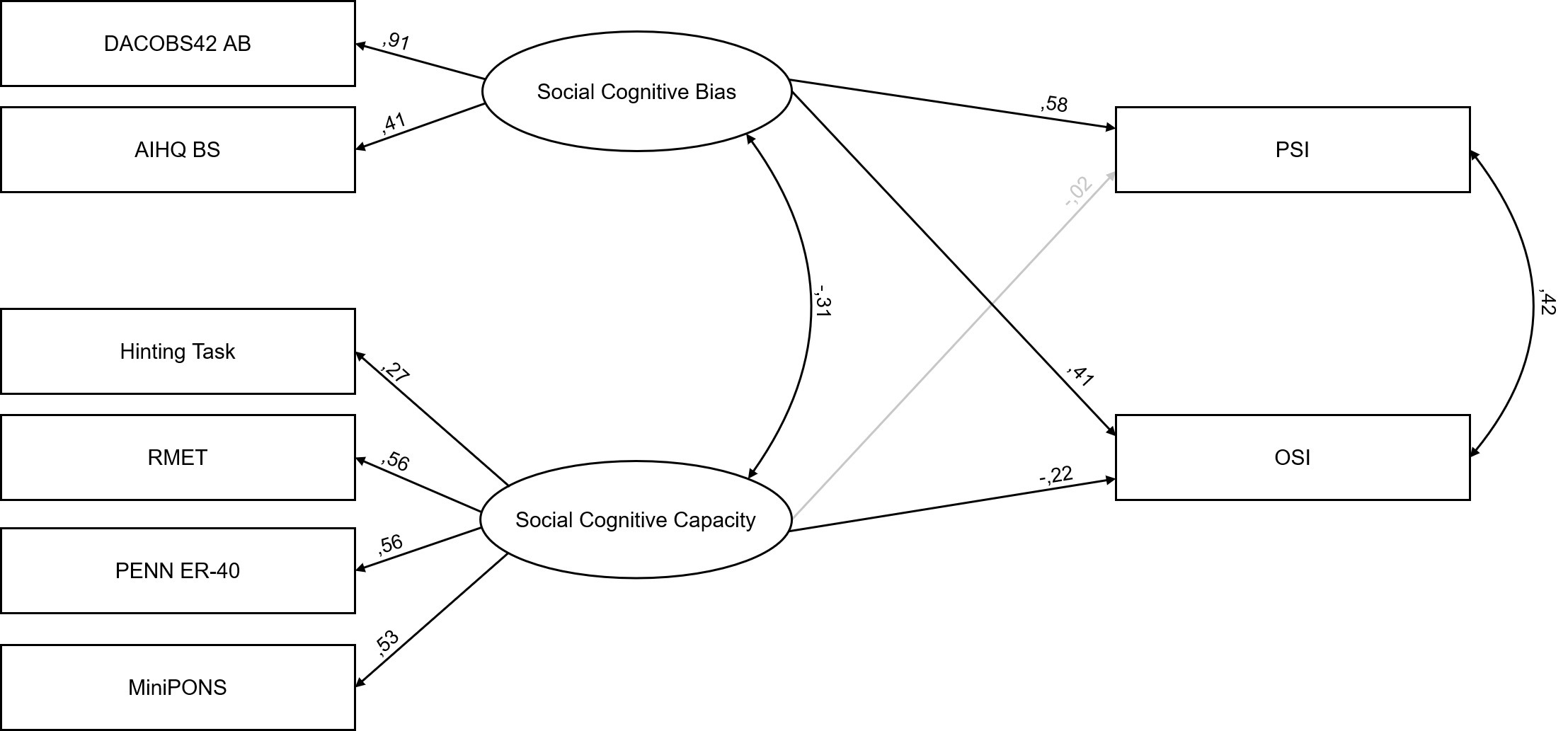

Supplement: nsae088_Supp [file nsae088_supp.zip › New folder/scan-24-139-File010.jpg]
